# First isolation of *Echinococcus granulosus sensu lato* genotype 7 in the archipelago of Cape Verde

**DOI:** 10.1017/S003118202300046X

**Published:** 2023-07

**Authors:** Lara Gonçalves Baptista, Teivi Laurimäe, Gillian Muchaamba, Laura Cathomas, Ana Lina Barros Olende, Iolanda Mata dos Santos, Ângela Lobo de Pina, Peter Deplazes

**Affiliations:** 1 Vetsuisse and Medical Faculty, Institute of Parasitology, University of Zurich, Zurich, Switzerland; 2 Bons Amigos Association, Praia, Cape Verde; 3 Department of Zoology, Institute of Ecology and Earth Sciences, University of Tartu, Tartu, Estonia; 4 Department of Livestock Services (DSP), General Directorate of Agriculture, Forestry and Livestock (DGASP), Ministry of Agriculture and Environment (MAA), Praia, Cape Verde

**Keywords:** Cape Verde, cattle, *Echinococcus granulosus sensu lato*, free-roaming dogs, G7, genotype 7, livestock, pig, zoonoses

## Abstract

There are no scientific data available on the occurrence of the *Echinococcus granulosus sensu lato* (*s.l.*) cluster in definitive hosts (domestic dogs), intermediate hosts (domestic livestock) nor humans in Cape Verde. In this pilot study, environmental dog fecal samples (*n* = 369) were collected around food markets, official slaughterhouses, as well as home and small business slaughter spots in 8 of the 9 inhabited islands from the Cape Verde archipelago, between June 2021 and March 2022. Additionally, during the same period, 40 cysts and tissue lesions were opportunistically collected from 5 islands, from locally slaughtered cattle (*n* = 7), goats (*n* = 2), sheep (*n* = 1) and pigs (*n* = 26). Genetic characterization by a multiplex polymerase chain reaction assay targeting the 12S rRNA gene confirmed the presence of *E. granulosus s.l.* in fecal and tissue material. In total, 17 cyst samples from Santiago (*n* = 9), Sal (*n* = 7) and São Vicente (*n* = 1) and 8 G6/G7-positive dog fecal samples from Santiago (*n* = 4) and Sal (*n* = 4) were identified as *E. granulosus s.l.* G7 by sequence analysis (*nad*2, *nad*5 and *nad*1 genes). This study discloses the transmission of *E. granulosus s.l.* G7, in pig, cattle and dog in Cape Verde.

## Graphical abstract

**Figure:**
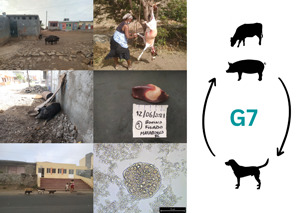

## Introduction

Cystic echinococcosis (CE) is a neglected parasitic zoonotic disease caused by tapeworm species and genotypes belonging to the *Echinococcus granulosus sensu lato* (*s.l.*) cluster, with differences in their life cycles, host ranges and geographical distribution (Deplazes *et al*., 2016; Romig *et al*., 2017). The *Echinococcus* taxonomy is complex and remains controversial (Thompson, 2017; Vuitton *et al*., 2020). Initially, 10 genotypes (G1–G10) were defined within *E. granulosus s.l.*, with a number of these genotypes later defined as discrete species. The currently widely accepted species within *E. granulosus s.l.* include the widespread *E. granulosus sensu stricto* (G1 and G3), *E. felidis*, *E. equinus* (G4), as well as *E. ortleppi* (G5). However, the species status of the *E. granulosus s.l.* genotype cluster G6–G8 and G10 remains under discussion (Vuitton *et al*., 2020). Some studies have suggested that the genetic differences taken together with biological, ecological and distribution range factors would warrant defining G6/G7 as a separate species (proposed name: *E. intermedius*) from the cervid G8/G10 (*E. canadensis*), while others have suggested the 4 genotypes belong to 1 species *E. canadensis* (G6–G8, G10) (Lymbery *et al*., 2015; Nakao *et al*., 2015; Laurimäe *et al*., 2018*a*, 2018*b*). Until a clear consensus is reached, for the purposes of this study, the genotypes G6 and G7 are referenced as *E. granulosus s.l.*

The parasite life cycle of *E. granulosus s.l.* includes 2 mammalian hosts. Carnivorous definitive hosts harbour the intestinal stages of *Echinococcus* spp. over several months without clinical signs. During the patent period, gravid proglottids are excreted in the feces, contaminating the environment with eggs, including soil, grass and water. After ingestion of eggs by intermediate or dead-end hosts, oncospheres hatch, invade the small intestine and migrate with the blood stream mainly to the liver and lungs (but also to other organs) and develop to the metacestode stage (*Echinococcus* cyst). The infected individuals may remain asymptomatic and undetected or may develop clinical symptoms, secondary to severe organ damage (Eckert and Deplazes, 2004; Romig *et al*., 2017).

CE is listed by the World Health Organization (WHO) as 1 of the 20 neglected tropical diseases that requires specific attention among poor and rural populations (Casulli, 2021) and it is listed by the World Organization for Animal Health (WOAH) as a disease that must be reported by members (WOAH, n.d.). It is considered a disease of public health importance with significant animal and human health consequences and socio-economic losses worldwide (Eckert *et al*., 2001; Budke *et al*., 2006). The highest prevalence of CE in animals and humans is found in countries of the temperate zones. CE is distributed worldwide and is widespread in Africa, where several genotypes have already been identified. However, data on prevalence, distribution and genetic diversity are broadly lacking in most of the African countries (Deplazes *et al*., 2017). Likewise, there are no scientific data available on the occurrence of *Echinococcus* spp. in dogs, intermediate hosts nor humans in Cape Verde.

The Cape Verde archipelago is part of Macaronesia region, located in the North Atlantic Ocean, 450–600 km west of Senegal and 1500 km southwest of the Canary islands. The archipelago is composed of 10 islands (of which 9 are inhabited) and 8 minor islets of volcanic origin (Ramalho, 2011). The main islands present a total surface area of 4033 km^2^ and are grouped in the northern group (Santo Antão, São Vicente, Santa Luzia and São Nicolau), the southern group (Santiago, Fogo and Brava) and the eastern group (Sal, Boavista and Maio) (Romeiras *et al*., 2014).

The islands were first discovered by Portuguese sailors around 1460, who found no evidence of previous human presence. Given the strategic location of the country on the routes that linked Europe, Africa and Brazil, the islands grew into an important trade centre. Cape Verde became independent from Portugal in 1975 and a parliamentary democracy was established in 1991. Presently, the population in the country is estimated to be around 500 000, with 76.8% of the population living in urban areas (INECV, 2022*a*). The largest and most populous island is Santiago, being also the most important agricultural and economic centre of the country. Praia (Santiago island) and Mindelo (São Vicente island) are the main cities of the country (INECV, 2022*a*) and Sal is the island that receives most of the tourists every year (INECV, 2022*b*).

The archipelago location on the Sahel area imposes an arid to semi-arid regime, with a temperate and warm climate, characterized by 2 seasons: a long dry season from November to July and a sparse and irregular rainy season from August to October (Ramalho, 2011). The prolonged drought periods, erosion and soil degradation, leading to desertification have been identified as the main constraints to agriculture, forestry and livestock development across the 10 islands of the archipelago (Monteiro *et al*., 2020). In particular, the feed deficit for the animals is a strong structural constraint to livestock development. According to the 2015 General Census of Agriculture (RGA), more than 60% of the population are still involved in livestock activities and dependent on this income [data not published, provided by the Department of Livestock Services (DSP)]; however, this sector contributes less than 2% to Cape Verde's gross domestic product (Bari, 2016)

Livestock farming is generally characterized by traditional smallholder subsistence farming systems, with low production and productivity, in small-size farms, where the animals roam free inside and nearby villages and settlements (Bari, 2016). In Cape Verde, there are around 80 000 livestock farms, with an average number of 2–3 animals per farm. According to the DSP from the Ministry of Agriculture and Environment (MAA), total livestock numbers in Cape Verde are approximately 108 000 goats, 67 000 pigs, 30 000 cattle and 13 000 sheep. Goats are mainly exploited for the milk and cheese production and pork is the most consumed type of meat in Cape Verde (Molina-Flores *et al*., 2020).

There is no registry on international import or export of live ruminants and swine in the last years on the World Trade Organization website (https://www.wto.org/english/thewto_e/acc_e/a1_capvert_e.htm). A great part of the livestock products consumption is satisfied by internal production, in particular meat from ruminants and swine (Bari, 2016). There are registries of livestock trade in-between islands. For example, in 2020, there are registries of the movement of 1704 cattle, 886 goats, 111 sheep and 26 pigs, most of the animals with origin in Santo Antão and Maio island; however, there is no information regarding the destination of the animals and some islands do not report at all the trades (data not published, provided by the DSP). Although there are legislations in place (Assembleia Nacional, 2018), there is still no national identification and marking system, making it impossible to keep records of origin, transport, births, deaths, slaughters and animal movements (data not published, provided by the DSP). In addition, there is a general lack of human resources, qualified personnel and equipment, and the absence of a duly equipped and functional laboratory which significantly impacts the capacity to perform the services and leaves a gap in the epidemiological surveillance. As a consequence, although there are specific legislations in place (Assembleia Nacional, 2013, 2020), most of the time the conditions are not met (data not published, provided by the DSP).

There is no available reliable information on the livestock market system. The farmers can either sell the live animals to middlemen or directly to slaughterers/butchers or sell the meat to the distributers/retailers (markets). The slaughter of animals is often done at home and small business slaughter spots, in open air and without inspection. Furthermore, most of the existing slaughterhouses are not working or work under extremely unhygienic and unsanitary conditions, are inadequately located (e.g. in the city centre) and poorly equipped (Bari, 2016). Presently, there are a total of 13 slaughterhouses functioning at a national level. The facilities are the responsibility of Town Halls. The operations are either carried out as a service by workers recruited by the Town Halls or each animal owner brings their own butcher. The inspection is the responsibility of the national veterinary services. However, despite the existence of an animal health inspection system, there is an absence of a monitoring system at all stages of the process and a lack of standardization quality control and certification protocol of animal origin products (data not published, provided by the DSP). It is estimated that around 80–90% of cattle slaughtering is performed at the slaughterhouses, while only 10% of pigs and less than 5% of goat and sheep slaughtering is controlled. The number of animals slaughtered outside controlled slaughterhouses and officially authorized slaughtering areas is significant and unknown (Bari, 2016). In 2021, there are registries of 3211 cattle, 2353 pigs, 551 goats and 60 sheep slaughtered at a national slaughterhouse (data not published, provided by the DSP).

The contact between livestock and dogs is frequent, as both populations roam free in Cape Verde. There are no available estimates for the number of dogs in the country. Antunes *et al*. (2015) estimated the dog population on Maio island to be of 531 individuals, based on a household survey and remotely sensed imagery, and obtained 1:12 average animal to human ratio, with higher ratios detected on more urbanized areas (1:8). Stray dogs are not considered as a big concern on Maio island where they are almost absent, which is in contrast to the rest of the islands, where free-roaming owned dogs represent the majority of the dog population and where the ratio of animal to human is expected to be higher, especially in the urban areas, where they can easily find essential resources available (water, food and shelter) (ICAM, 2019). Despite the effort of local entities and non-governmental animal welfare organizations working on dog population management programmes in Cape Verde, the population of stray dogs is still uncontrolled on most of the islands. The presence of stray dogs can have a negative impact on public health, due to the close contact between domestic animals, livestock and humans.

The presence of free-roaming livestock with access to dog feces, as well as the environment that is contaminated with parasite eggs, coupled with the non-official practice of animal slaughtering and insufficient hygiene in slaughterhouses are all factors that are present in Cape Verde, which could possibly facilitate the transmission of *Echinococcus* spp. As well as the lack of appropriate animal health surveillance and access of dogs to offal, adding to the high number of free-roaming, un-dewormed dogs with uncontrolled health status, living close to humans and livestock. Similar situations as described here have been identified as the main drivers for CE transmission in other countries (Deplazes *et al*., 2017). The pre-conditions for the transmission of *E. granulosus s.l.* between domestic dogs and a variety of potential domestic herbivorous mammals acting as intermediate hosts are all present in Cape Verde. As there are no wild carnivores or herbivores present in Cape Verde, the *Echinococcus* cycle would be restricted to domestic animals.

Scientific literature on the presence of *E. granulosus s.l.* or reports of CE in Cape Verde are absent, and to the best of our knowledge, to date no systematic studies on the occurrence of the parasite and the disease on the different islands have been performed. Slaughterhouse records have not shown the presence of cysts or tissue lesions in livestock and human cases have not been reported (only anecdotal reports). The aim of this study was to investigate the possible occurrence and transmission of *E. granulosus s.l.* in Cape Verde.

## Materials and methods

### Collection of environmental dog fecal sample

**Figure 1.**
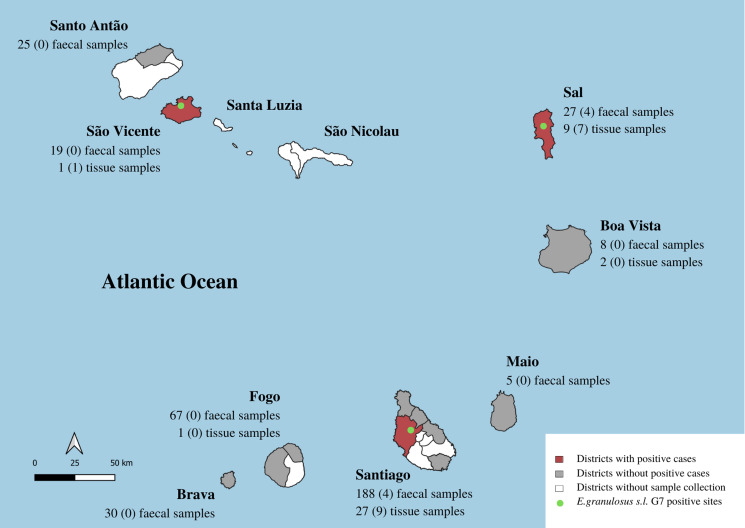
Map of Cape Verde archipelago, with indication of the geographic origin (islands and districts) with positive cases (red area), with samples collected but no confirmed positive cases (grey area) and where sample collection was not carried out (white area). The geographic distribution of *Echinococcus granulosus s.l.* G7 isolated from dog fecal samples and cysts and/or lesions (tissue samples) from livestock is shown by the green dots. The total number of samples collected per island and the number of positive cases of *Echinococcus granulosus s.l.* G7 (in brackets) are indicated.

All in all, 369 dog fecal samples were collected from the environment in 8 of the 9 inhabited islands of Cape Verde: namely Santiago, Fogo, Brava, Sal, Maio, Boa Vista, São Vicente and Santo Antão, from June 2021 to February 2022 (Table 1 and Fig. 1). The fecal samples were collected around areas considered at risk (markets, official slaughterhouses, other slaughter spots) where dogs could have access to raw offal. The samples were selected merely based on morphological features (e.g. size, contents), which might have enabled the inclusion of human and/or cat fecal samples in a few cases.

**Table 1.** Collection of dog fecal samples, and cysts and tissue lesions from pigs, cattle, goats and sheep

Island	District	No. of fecal samples collected	Cysts and tissue lesions	
			Samples collected	Hosts
Santiago	Santa Catarina	71	26	13 Pigs 7 Cattle 1 Goat 1 Sheep
	Praia	71	1	1 Pig
	Santa Cruz	13	0	N/A
	São Miguel	27	0	N/A
	Tarrafal	6	0	N/A
Brava	Brava	30	0	N/A
Fogo	São Filipe	26	1	1 Pig
	Mosteiros	41	0	N/A
Sal	Sal	27	9	9 Pig
Maio	Maio	5	0	N/A
Boa Vista	Boa Vista	8	2	1 Pig 1 Goat
São Vicente	São Vicente	19	1	1 Pig
Santo Antão	Ribeira Grande	25	0	N/A
**Total**		**369**	**40**	**36**

The majority of the dog fecal samples (*n* = 295) were shipped for further examination to the Institute of Parasitology, University of Zurich (IPZ-UZH); with exception of 74 samples, which were sieved at Cape Verde, at DSP laboratory with personnel wearing protective clothing including overalls, gloves, aprons, head and shoes covers; and shipped as sediments. For safety reasons, all feces and sediments were stored at −80°C at IPZ-UZH for at least 3 days to inactivate taeniid eggs and then subsequently at −20°C until further use.

### Coprological examination

Two grams of feces were used for coprological examination with sequential sieving for taeniid egg isolation as described in Mathis *et al*. (1996), and modified using floatation with sugar solution and using polyethylene terephthalate (PET) bottles in a 1-way system to prevent DNA contamination (Sharma *et al*., 2021). Taeniid eggs were identified on an inverted microscope in flat-sided tubes. All samples were centrifuged at 200 ***g*** for 10 min and kept in 1.5 mL tubes at −20°C. Using an alkaline lysis method, as described in Bucher *et al*. (2021), genomic DNA was extracted from all the microscopically taeniid egg-positive samples and randomly selected 40% of the negative samples from each district, to document the sensitivity of the microscopic evaluation. Briefly, eggs were treated with 0.2 M NaOH, incubated at +95°C for 10 min and then 1 part of the supernatant was diluted in 50 parts of 100 mm Tris-HCl which was used as a template for the polymerase chain reaction (PCR).

### Collection of cysts and tissue lesions

A total of 40 putative metacestodes including typical *Echinococcus* cysts but also other cysts and tissue lesions with no morphological cyst formation were opportunistically collected from 5 islands (Santiago, Sal, Fogo, Boa Vista and São Vicente) from June 2021 to February 2022 (Table 1 and Fig. 1).

The samples were dissected from domestic animals during slaughter or during meat inspection at the markets. Cysts were obtained from cattle (*n* = 7), goats (*n* = 2), sheep (*n* = 1) and pigs (*n* = 26). A single sample was collected per each host animal, except in one instance, where 5 cysts were collected from the same pig liver. The cysts were collected from heart (*n* = 1), intestine (*n* = 1), kidney (*n* = 2), liver (*n* = 31), lung (*n* = 1) and *omentum* (*n* = 4) (Supplementary Table S1). The samples were stored in 70% ethanol before being shipped to IPZ-UZH for molecular examination.

### Examination of cysts and tissue lesions

All cysts and tissue lesions were examined before DNA extraction. The fluid from each cyst was aspirated using a syringe, and the sediment was observed microscopically for the presence of protoscoleces or taeniid hooks. In the absence of these, the whole cyst or lesion was aseptically dissected, and the germinal layer scraped for further microscopic examination as previously described (Gareh *et al*., 2021). Cysts were classified according to the presence of fluid and protoscoleces as either sterile, fertile or calcified (Table 3). The protoscoleces or germinal layer of each cyst or lesion was washed 3 times with phosphate-buffered saline for later use in DNA extraction using a commercial QiaAmp DNA Mini kit (Qiagen, Hilden, Germany) following the manufacturer's protocol.

### Detection of taeniid DNA in fecal and tissue material

A multiplex PCR using primers detecting *E. granulosus s.l.*, *E. multilocularis*, *Taenia* species and a large range of other cestode species (including Mesocestoides and Dipylidium), targeting the mitochondrial 12S rRNA and NADH dehydrogenase subunit 1 (*nad*1) gene regions, was used to amplify the DNA from the cyst and fecal material (Trachsel *et al*., 2007). The PCR amplicons were analysed on a 2% agarose gel, thereafter positive amplicons were purified using a MinElute PCR Purification Kit (Qiagen) as per the manufacturer's instructions. Sequencing was performed at Microsynth AG (Balgach, Switzerland). The quality of the sequences was checked, and misread nucleotides were manually corrected, followed by sequence alignment and trimming using Geneious Prime Software (2021). Sequence homology was assessed by comparison with available sequences in the NCBI Nucleotide Blast algorithm (https://blast.ncbi.nlm.nih.gov/).

### Genetic characterization of *E. granulosus sensu lato* isolates

As the primers described in Trachsel *et al*. (2007) could not distinguish between *E. granulosus s.l.* genotypes G6, G7 and G10 (see e.g. Laurimäe *et al*., 2023), samples that were determined as positive for *E. granulosus s.l.* with the multiplex PCR were subjected to further molecular characterization using primers targeting the mitochondrial NADH dehydrogenase subunit 2 (*nad*2) and NADH dehydrogenase subunit 5 (*nad*5) genes (Kinkar *et al*., 2018; Laurimäe *et al*., 2019). The PCR conditions were essentially as described in Laurimäe *et al*. (2019) with minor modifications. The PCR reactions were carried out in 50 *μ*L, with 0.2 *μ*m of each primer and 5 *μ*L of template DNA (<1 *μ*g) using the Qiagen Multiplex PCR kit (Qiagen). The touch-down PCR conditions were as follows: 95°C for 15 min, followed by 10 cycles of 94°C for 30 s, 55°C for 45 s (annealing temperature progressively reduced by 0.5°C in each cycle) and 72°C for 90 s; followed by 30 cycles of 94°C for 30 s, 50°C for 45 s, 72°C for 90 s; and finishing with a final elongation step at 72°C for 5 min.

In order to allow for additional comparisons of *E. granulosus s.l.* sequence data originating from other countries in Africa, particularly in comparison of available G7 sequences, the widely applied mitochondrial NADH dehydrogenase 1 (*nad*1) gene was amplified using previously described primers (Hüttner *et al*., 2008; Aschenborn *et al*., 2022). The PCR was carried out as described above. PCR conditions for the *nad*1 primers described in Hüttner *et al*. (2008) and Aschenborn *et al*. (2022) were as follows: initial denaturation at 95°C for 15 min, 37 cycles of denaturation at 94°C for 30 s, annealing at 55°C for 30 s, extension at 72°C for 60 s and final extension at 72°C for 10 min.

Positive amplicons were confirmed on 2% agarose gel, purified using a commercial kit (MinElute PCR Purification Kit, Hilden, Germany) and sent for sequencing at Microsynth. The sequence data were analysed for errors, aligned and trimmed in Geneious Prime Software, 2021.

### Phylogenetic network

To allow for sequence comparison and accurate genotype determination of the *E. granulosus s.l.* samples, 3 datasets were created: (i) concatenated *nad*2 (681 bp) and *nad*5 (614 bp); (ii) *nad*1 (882 bp); (iii) concatenated *nad*2 (681 bp), *nad*5 (614 bp) and *nad*1 (513 bp). Initial sequence confirmation was done using the NCBI Nucleotide Blast algorithm (https://blast.ncbi.nlm.nih.gov/), and further identification of genotype achieved by comparison with reference sequences in a median joining phylogenetic network analysis using the Network Software 10.2 (Fluxus Technology Ltd, Colchester, United Kingdom), with indels and point mutations considered. Reference sequences were downloaded from Mendeley and from NCBI GenBank (Supplementary Table S2).

## Results

### Coprological examination

Collectively on all islands, 32 (8.7%) out of 369 dog fecal samples collected were positive by microscopic observation and/or multiplex PCR for taeniids. The sensitivity for the detection was 84.4% (95% CI 67.2–94.72%) and 84.2% (95% CI 67.8–94.0%) using microscopic observation and multiplex PCR, respectively. Eight (2.2%) fecal samples were positive for *E. granulosus s.l.* yielding a 117 bp amplicon, while 24 (6.5%) samples were positive for *Taenia* sp. and other cestodes (267 bp). After sequencing of the 267 bp amplicon, the NCBI Nucleotide BLAST confirmed the identity of 22 samples as *T. hydatigena*; 1 sample was *Hydatigera taeniaeformis*, whereas 1 remained inconclusive (*Taenia* sp.) (Supplementary Table S3). Sequence analysis of the 117 bp amplicons positive for *E. granulosus s.l.* sequences revealed that all 8 samples were *E. granulosus s.l.* G6/G7. Four of these samples were originally from the island of Santiago, while the other 4 were from the island of Sal (Table 2 and Fig. 1).

**Table 2.** Origin, microscopic examination and molecular species identification of taeniid eggs from environmental dog fecal samples collected from different islands in Cape Verde

Origin	District	Number of samples collected	Microscopic detection of taeniid eggs	Multiplex PCR^a^ positive/total tested	Sequencing (*n* – number confirmed)
Santiago	Santa Catarina	71	8 Positive 63 Negative^b^	6/8 1/26	*E. granulosus s.l.*^c^ (4), *Taenia hydatigena* (2) *Hydatigera taeniaeformis* (1)
	Praia	71	9 Positive 62 Negative^b^	7/9 4/25	*T. hydatigena* (10) *Taenia* sp. (1)
	Santa Cruz	13	2 Positive 11 Negative^b^	2/2 0/4	*T. hydatigena* (2)
	São Miguel	27	1 Positive 26 Negative^b^	1/1 0/10	*T. hydatigena* (1)
	Tarrafal	6	0 Positive 6 Negative^b^	0/0 0/4	–
Brava	Brava	30	0 Positive 30 Negative^b^	0/0 0/16	–
Fogo	São Filipe	26	0 Positive 26 Negative^b^	0/0 0/12	–
	Mosteiros	41	2 Positive 39 Negative^b^	2/2 0/14	*T. hydatigena* (2)
Sal	Sal	27	5 Positive 22 Negative^b^	4/5 0/8	*E. granulosus s.l.*^c^ (4)
Maio	Maio	5	0 Positive 5 Negative^b^	0/0 0/2	–
Boa vista	Boa Vista	8	1 Positive 7 Negative^b^	1/1 0/4	*T. hydatigena* (1)
São Vicente	São Vicente	19	2 Positive 17 Negative^b^	2/2 0/7	*T. hydatigena* (2)
Santo Antão	Ribeira Grande	25	3 Positive 22 Negative^b^	2/3 0/8	*T. hydatigena* (2)
**Total**		**369**	**33 Positive** **336 Negative**^b^	**27/33** **5/140**	

a PCR primers and conditions ran as described by Trachsel *et al*. (2007).

b DNA extraction was performed on 40% of the negative samples.

c Further genetics characterized using nad2, nad5 and nad1 primer combination, refer to Table 3.

### Examination of cysts and tissue lesions

From the 40 cysts and lesions analysed, 7 (17.5%) samples failed to amplify and 16 (40%) were positive for *Taenia* spp. (Supplementary Table S3), revealing that 15 of these sequences had 100% homology to *T. hydatigena* with available sequences from GenBank (accession number AB027135). The *T. hydatigena*-positive samples included 4 from cattle, 1 from sheep, 2 from goats and 8 from pigs. One sample, from a pig, remained inconclusive (*Taenia* sp.).

All *Echinococcus* cyst samples (*n* = 17; 42.5%) were positive for *E. granulosus s.l* with the multiplex PCR and sequencing results of 12S rRNA (117 bp) indicated all samples were G6/G7 (data not shown). Nine of these samples originated from the island of Santiago, 7 from the island of Sal and 1 from the island of São Vicente (Table 3 and Fig. 1). From all, 94% (16/17) of the cysts were reported in the liver with a fertility rate of 35%. One cyst from cattle and 5 from pigs were fertile, while 10 others from pigs were sterile, and 1 cyst from a pig was calcified (Table 3).

**Table 3.** Sample ID, designated haplotype name on the phylogenetic networks, origin (island, district, city, local and place of collection), host species, sample type and cyst condition for *E. granulosus s.l.* G7 samples identified from tissue cysts and fecal samples in Cape Verde. Genotype confirmed by concatenated mitochondrial gene sequences of *nad*5 and n*ad*2.

Sample ID	nad2 + nad5 haplotype	nad1 haplotype	Origin (island)	District, city, local	Place of collection	Host, organ	Sample type	Cyst condition
1	HAP1	HAP2	Santiago	Santa Catarina, Assomada, city centre	Slaughterhouse	Cattle, liver	Metacestode	Fertile
2	HAP1	HAP2	Santiago	Santa Catarina, Assomada, city centre	Municipal market/butcher	Pig, liver	Metacestode	Sterile
3	N/A	HAP2	Santiago	Santa Catarina, Assomada, city centre	Slaughterhouse	Pig, lung	Metacestode	Fertile
4^a^	HAP1	HAP2	Santiago	Santa Catarina, Assomada, City centre	Slaughterhouse	Pig, liver	Metacestode	Sterile
4a^a^	HAP1	N/A	Santiago	Santa Catarina, Assomada, city centre	Slaughterhouse	Pig, liver	Metacestode	Sterile
4b^a^	HAP1	N/A	Santiago	Santa Catarina, Assomada, city centre	Slaughterhouse	Pig, liver	Metacestode	Sterile
4c^a^	HAP1	N/A	Santiago	Santa Catarina, Assomada, city centre	Slaughterhouse	Pig, liver	Metacestode	Sterile
4d^a^	HAP1	N/A	Santiago	Santa Catarina, Assomada, city centre	Slaughterhouse	Pig, liver	Metacestode	Sterile
5	HAP1	HAP2	Santiago	Santa Catarina, Assomada, city centre	Slaughterhouse	Pig, liver	Metacestode	Sterile
6	HAP1	HAP2	Sal	Sal, Espargos, city centre	Slaughterhouse	Pig, liver	Metacestode	Fertile
8	N/A	HAP2	Santiago	Santa Catarina, Assomada, Cumben	Around home slaughter spot	Dog	Fecal	N/A
9	HAP1	N/A	Santiago	Santa Catarina, Assomada, Cumben	Around home slaughter spot	Dog	Fecal	N/A
10	HAP1	N/A	Santiago	Santa Catarina, Assomada, Cumben	Around home slaughter spot	Dog	Fecal	N/A
11	HAP1	N/A	Santiago	Santa Catarina, Assomada, Cumben	Around home slaughter spot	Dog	Fecal	N/A
12	HAP1	N/A	Sal	Sal, Pedra Lume, Alto Solarino	Around home slaughter spot	Dog	Fecal	N/A
13	HAP1	N/A	Sal	Sal, Espargos, Terra Boa	Around home slaughter spot	Dog	Fecal	N/A
14	HAP1	N/A	Sal	Sal, Espargos, Terra Boa	Around home slaughter spot	Dog	Fecal	N/A
15	HAP1	N/A	Sal	Sal, Espargos, Alto Santa Cruz	Around home slaughter spot	Dog	Fecal	N/A
16	HAP1	N/A	São Vicente	São Vicente, Mindelo, Ribeira de Craquinha	Slaughterhouse	Pig, kidney	Metacestode	Calcified
17	HAP1	HAP2	Sal	Sal, Espargos, city centre	Slaughterhouse	Pig, liver	Metacestode	Fertile
18	HAP1	N/A	Sal	Sal, Espargos, city centre	Slaughterhouse	Pig, liver	Metacestode	Fertile
19	HAP1	N/A	Sal	Sal, Espargos, city centre	Slaughterhouse	Pig, liver	Metacestode	Fertile
20	HAP1	N/A	Sal	Sal, Espargos, city centre	Slaughterhouse	Pig, liver	Metacestode	Sterile
21	HAP1	N/A	Sal	Sal, Espargos, city centre	Slaughterhouse	Pig, liver	Metacestode	Sterile
22	HAP1	N/A	Sal	Sal, Espargos, city centre	Slaughterhouse	Pig, liver	Metacestode	Sterile

N/A, not applicable.

a Different cysts from the sample animal.

All *Echinococcus*-positive samples were classified as metacestodes. However, as data concerning cyst/tissue lesion description are missing, it is not possible to conclude on the sample structure of the other samples.

### Genetic characterization of *E. granulosus s.l.* isolates from feces and metacestodes

PCR amplification of the *nad*2 gene yielded a product of 781 bp, while that of the *nad*5 gene yielded a 759 bp fragment. Following quality control and manual trimming of sequences, the sequences submitted to further analysis were 681 and 614 bp for *nad*2 and *nad*5, respectively. Of the 8 fecal samples determined as *E. granulosus s.l.* based on the 12S rRNA (117 bp) multiplex PCR, the *nad*2 and *nad*5 sequences were successfully obtained for a total of 7 fecal samples (Supplementary Table S2). Whereas out of 17 cyst and tissue lesions samples determined as *E. granulosus s.l.* based on the multiplex PCR, the *nad*2 and *nad*5 gene fragments were successfully amplified for 16 samples obtained from 12 intermediate host animals. As in case of 1 animal (ID4) multiple cysts were found that showed identical sequences, for phylogenetic network analysis only sequence data from 1 cyst per animal was included (total *n* = 12) (Supplementary Table S2). NCBI Nucleotide BLAST revealed a 100% homology to G7 (accession number MH301004) for all Cape Verde samples (both cyst and fecal material) based on the *nad*2 and *nad*5 sequence data (Supplementary Table S2).

Furthermore, the *nad*1 (882 bp) gene fragment was successfully amplified for 1 fecal sample and 7 cyst isolates (Supplementary Table S2). This included 1 cyst sample (ID3) and 1 fecal sample (ID8) that both previously failed to amplify with the *nad*2 and *nad*5 primers. Genotypic identity for these 2 samples was determined solely based on the *nad*1 gene. NCBI Nucleotide BLAST revealed a 100% homology with G7.

### Phylogenetic network

**Figure 2.**
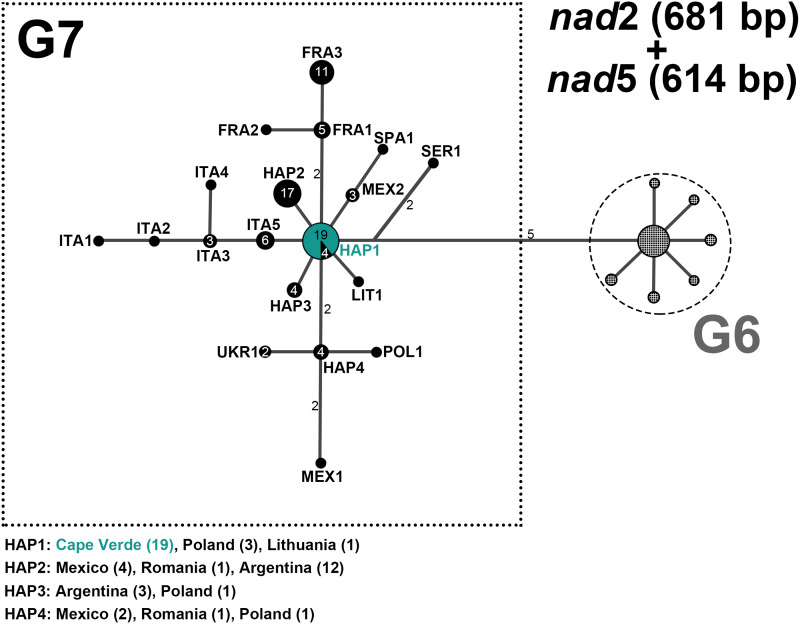
Median-joining network of *Echinococcus granulosus s.l.* G7 isolates from Cape Verde and respective G6–G7 reference sequences from GenBank based on gene fragments of concatenated NADH dehydrogenase subunits 2 (*nad*2, 681 bp) and 5 (*nad*5, 614 bp). Cape Verde sequences of the current study obtained from tissue (*n* = 13) and fecal samples (*n* = 7) are depicted in green. References from GenBank of genotype G6 are represented schematically, and references of G7 are depicted in black. Numbers inside the circles represent the number of identical sequences within the respective haplotype; numbers beside the lines represent the number of mutations. Haplotype names are designated as 3 letter abbreviations [HAP, haplotypes representing samples originating from different countries; FRA, France (Corsica); ITA, Italy; LIT, Lithuania; MEX, Mexico; POL, Poland; SER, Serbia; SPA, Spain; UKR, Ukraine].

**Figure 3.**
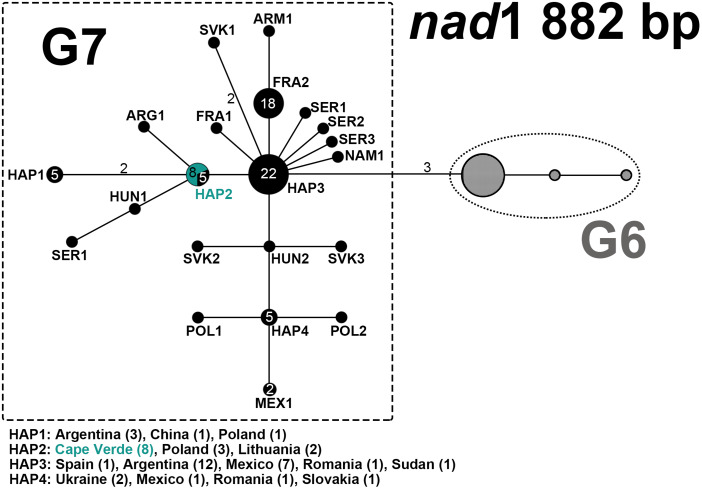
Median-joining network of *Echinococcus granulosus s.l.* G7 isolates from Cape Verde and respective G6–G7 reference sequences from GenBank based on NADH dehydrogenase subunit 1 (*nad*1, 882 bp) gene fragment. Cape Verde sequences of the current study obtained from tissue (*n* = 7) and fecal samples (*n* = 1) are depicted in green. References from GenBank of genotype G6 are represented schematically, and references of G7 are depicted in black. Numbers inside the circles represent the number of identical sequences within the respective haplotype; numbers beside the lines represent the number of mutations. Haplotype names are designated as 3 letter abbreviations [HAP, haplotypes representing samples originating from different countries; ARG, Argentina; ARM, Armenia; FRA, France (Corsica); HUN, Hungary; MEX, Mexico; NAM, Namibia; POL, Poland; SER, Serbia; SVK, Slovakia].

The phylogenetic network analysis of *nad*2 and *nad*5 from tissue (*n* = 12) and fecal (*n* = 7) samples revealed that successfully sequenced Cape Verde samples (*n* = 19) clustered into a centrally placed haplotype HAP1 (Fig. 2), showing 100% homology to an isolate from Lithuania (isolated by Professor Mindaugas Šarkūnas), sequenced during the course of the current study (ID7; Supplementary Table S2) and GenBank reference sequences originating from Poland (*n* = 3; Supplementary Table S2). Similarly, the *nad*1 gene phylogenetic network analysis (Fig. 3) placed the Cape Verde samples (*n* = 8) in 1 haplotype HAP2 together with 2 isolates from Lithuania (one GenBank reference, the other sequenced in the current study), and 3 reference sequences from Poland. Based on the *nad*1 gene, the other previously determined G7 sequence available from Africa (NAM1, Namibia) was separated from the Cape Verde samples by 2 mutations. Additionally, the phylogenetic network analysis of concatenated *nad*2, *nad*5 and *nad*1 (Supplementary Fig. S1) placed the Cape Verde samples (*n* = 5) in 1 haplotype HAP1 together with 1 isolate from Lithuania and 3 reference sequences from Poland.

## Discussion

The present study confirms the occurrence of *E. granulosus s.l.* G7 in Cape Verde, disclosing that this genotype, with the potential of zoonotic transmission, is circulating in the country. Although G7 was the only genotype found in this study, at this stage we cannot exclude the possibility that other genotypes of *E. granulosus s.l.* occur in Cape Verde, as the conditions for other parasite cycles are present (e.g. home slaughter of goats). However, the identification of G7 in all samples obtained from intermediate hosts and of G6/G7 in 8 *Echinococcus* egg isolates from dogs (7 confirmed being G7 based on *nad*2 and *nad*5) documents that G7 is the most abundant genotype contaminating the environments investigated in the current study.

The location of 94% of the positive cysts in the liver of the intermediate hosts is in agreement with the finding from Ohiolei *et al*. (2022) that suggested that *E. granulosus s.l.* G7 might have a preference for this organ. Furthermore, the high number of fertile cysts from pigs indicates they are the main hosts contributing to the parasite transmission. Although the single cyst found from cattle was fertile, the contribution to the parasite transmission would likely be considered insignificant as the genotype is more commonly transmitted in the dog–pig cycle (Romig *et al*., 2017). However, G7 has been described in other intermediate hosts such as cattle, domestic goats, oryx antelope (*Oryx gazella*) and wild boar (*Sus scrofa*) (Varcasia *et al*., 2007; Beato *et al*., 2013; Onac *et al*., 2013; Umhang *et al*., 2014; Aschenborn *et al*., 2022). Here, we confirmed the presence in cattle, but we cannot exclude the presence in other intermediate host species, as the places available for investigation during the course of the study mainly slaughtered cattle and pigs.

The parasite was confirmed on the islands of Santiago, Sal and São Vicente, and is likely transmitted focally, since it was detected in environmental samples and fertile cysts were identified from livestock (mainly pigs). This is furthermore supported by the genetic analysis where the sequences from both environmental samples and livestock tissue were shown to be identical. All in all, the positive *E. granulosus s.l.* G7 fecal samples were from 4 areas in different islands where home slaughter is practiced, in Cumben (Assomada city, Santa Catarina district, Santiago island), in Pedra Lume locality (Sal district, Sal island) and in Terra Boa and Alto Santa Cruz (Espargos city, Sal district, Sal island). The positive cysts were found in Assomada city centre, Espargos city centre and also in Ribeira Craquinha (Mindelo city, São Vicente district, São Vicente island). However, the samples of the current study were collected during inspection at the municipal slaughterhouses and butchers at the respective islands. All the environmental samples collected around the slaughterhouses were negative, and unfortunately, during the course of this investigation, it was not possible to inspect the slaughter of any livestock in the home slaughter spots near the places with positive environmental samples. Moreover, the presence of *T. hydatigena* in 22 dog fecal samples, together with the finding of *E. granulosus s.l.* described in this study, shows that some dogs are actively being fed or have access to livestock offal and raw meat. This was also confirmed by butchers and dog owners informally questioned during the sample collection.

From 1 fecal sample of a dog, DNA of *H. taeniaeformis* was detected. This tapeworm parasitizes mainly felids but has seldomly also been found in canids too (e.g. Deplazes, personal communication). However, as the host species origin was determined based on morphological characteristics of the fecal sample, we cannot exclude in this case that the fecal sample could have originated from a cat instead.

The G7 genotype has a wide distribution range including Europe, where the main endemic areas are the Baltic states, Poland and Eastern Europe, whereas elsewhere in Central Europe, Spain (Daniel Mwambete *et al*., 2004), Italy (Genchi *et al*., 2021), Portugal (Guerra *et al*., 2013) and France (Corsica) (Umhang *et al*., 2020), G7 is distributed predominantly only focally (Deplazes *et al*., 2017; Casulli *et al*., 2022). It has also been identified in the Middle East, Mexico and Argentina (Kamenetzky *et al*., 2002; Villalobos *et al*., 2007; Borhani *et al*., 2021), and more recently, in the African Continent, in Namibia (Aschenborn *et al*., 2022). This genotype is mostly present in pig-rearing regions (Romig *et al*., 2017), with predominant smallholder traditional pig farming and with non-supervised slaughter, as documented in Lithuania (Bružinskaite *et al*., 2009) and Poland (Dybicz *et al*., 2013). Interestingly, according to mitochondrial *nad*2 and *nad*5 sequences of the current study, all Cape Verde isolates showed 100% sequence homology and clustered into 1 haplotype together with available sequences from Poland and Lithuania. This may suggest that the genotype found in Cape Verde, was most probably introduced from Europe, either by an imported infected livestock or domestic dogs in the past. Based on the history of the colonization of the islands, the most probable origin of the pig population derives from Portugal. Unfortunately, only short fragments of the mitochondrial cytochrome c oxidase subunit 1 (*cox*1) gene were available from Portugal. However, as recent studies have raised concerns about the accuracy of genotype determination based on *cox*1 for *E. granulosus s.l.* G6 and G7, other markers were chosen for the current study that were previously shown to allow for a clear distinction between the closely related genotypes (Laurimäe *et al*., 2019). Furthermore, as no historical data are available on pig liver parasites in Cape Verde, we cannot conclude when this parasite has been introduced to the islands.

The zoonotic potential of G7 genotype has been documented alongside the significant number of human cases, e.g. in Lithuania (Marcinkute *et al*., 2015). Nevertheless, according to the surgeons from the Dr Agostinho Neto Hospital in Praia (the biggest hospital in the country), CE in humans in Cape Verde is considered rare and no cases have been reported in the last 3 years (personal communication).

One of the limitations of the study was the lack of animal identification and tracking system, which does not allow to trace back the exact origin of the animals. The biggest livestock market in the country takes place every week in the city of Assomada (Santiago islands). Animals originating from all the islands are taken to the market to be sold and then transported again to any other district or island. It is therefore impossible to make conclusions on the focal areas of transmission. Furthermore, as there are no realistic records of the number of animals slaughtered in the country and it is not routine to report or collect and store cysts or pathological tissue lesions during inspection, it is not possible to conclude on prevalence or incidence in the study. Likewise, regarding the environmental samples, as some of the positive ones were collected in the same spot, we cannot exclude that they may belong to the same individual, thus it is also not possible to infer on prevalence or incidence of the parasite on the definitive host population. However, as no cases occurred in humans, it may perhaps be low. It might also be a good practice that the streets in Cape Verde are cleaned daily by Town Halls workers.

There are recognized challenges and difficulties on the elimination of CE (Craig and Larrieu, 2006). However, the G7 genotype is particularly susceptible to veterinary public health activities as the parasite is predominantly transmitted in a dog–pig cycle with a well-defined and mostly urban or even restricted transmission on infected farms (Šarkūnas *et al*., 2019). The few positive samples found in the study together with the lack of reports of human cases appear to indicate that CE might not be a serious animal and human health concern in Cape Verde and could realistically be controlled by systematic and coordinated implementation of One Health strategies. For this reason, it is recommended to improve the veterinary public health conditions at slaughterhouses and other slaughter spots including, meat inspection, correct offal disposal and carcasses destruction. In addition, the development of an integrated national dog population management programme is suggested to promote dog population control and regular deworming programmes.

## Data Availability

GenBank accession numbers: OP976149–OP976205

## Supplementary material

For supplementary material accompanying this paper visit https://doi.org/10.1017/S003118202300046X. click here to view supplementary material
