# Supplementary material for: First isolation of Echinococcus granulosus sensu lato genotype 7 in the archipelago of Cape Verde
Source: Parasitology. 2023 May 17;150(8):734–43. doi: 10.1017/S003118202300046X (PMC10410394; doi:10.1017/S003118202300046X)
Supplement: Supplementary file 1 [file S003118202300046Xsup001.docx]

First isolation of *Echinococcus granulosus sensu lato* Genotype 7 in the Archipelago of Cape Verde

Lara Sofia Gonçalves Baptista^1,2*^, Teivi Laurimäe^1,3^, Gillian Muchaamba^1^, Laura Cathomas^1^, Ana Lina Pereira de Barros Olende^4^, Iolanda da Mata dos Santos^4^, Ângela Cristina Lobo de Pina^4^, Peter Deplazes^1*^

**Supplementary materials**

Table S1 – Number of cysts and pathological lesions collected with detailed host species and organs affected. One sample was collected from each host, except in one case with five cysts collected from the same pig liver.

| **Animals** | **Organs affected** | **Nr of cysts and lesions** |
| --- | --- | --- |
| Cattle | Heart | 1 |
|  | Liver | 5 |
|  | Intestine | 1 |
| Goats | Liver | 1 |
|  | *Omentum* | 1 |
| Sheep | *Omentum* | 1 |
| Pigs | Liver | 25 |
|  | Lungs | 1 |
|  | Kidney | 2 |
|  | *Omentum* | 2 |
| TOTAL |  | 40 |

Table S2 - Sequences of the current study, and reference sequences from GenBank included in the respective datasets, their sample IDs, origin, hosts, genotypes, and respective assigned haplotype names.

| **Sample ID** | **Country** | **Host** | **Genotype** | **Dataset (i)**  **nad2+nad5 haplotype (Fig.2)** | **Dataset (ii)**  **nad1 haplotype (Fig.3)** | **Dataset (iii)**  **nad2+nad5**  **+nad1 haplotype (Fig. S1)** | **Whole mitogenome accession** | **GenBank**  **accession (nad2)** | **GenBank**  **accession2 (nad5)** | **GenBank accession3 (nad1)** |
| --- | --- | --- | --- | --- | --- | --- | --- | --- | --- | --- |
| 1 | Cape Verde | cow | G7 | HAP1 | HAP2 | HAP1 | N/A | OP976149 | OP976173 | OP976197 |
| 2 | Cape Verde | pig | G7 | HAP1 | HAP2 | HAP1 | N/A | OP976150 | OP976174 | OP976198 |
| *3* | Cape Verde | pig | G7 | N/A | HAP2 | N/A | N/A | N/A | N/A | OP976199 |
| 4 | Cape Verde | pig | G7 | HAP1 | HAP2 | HAP1 | N/A | OP976151 | OP976175 | OP976200 |
| 4a | Cape Verde | pig | G7 | HAP1* | N/A | N/A | N/A | OP976152 | OP976176 | N/A |
| 4b | Cape Verde | pig | G7 | HAP1* | N/A | N/A | N/A | OP976153 | OP976177 | N/A |
| 4c | Cape Verde | pig | G7 | HAP1* | N/A | N/A | N/A | OP976154 | OP976178 | N/A |
| 4d | Cape Verde | pig | G7 | HAP1* | N/A | N/A | N/A | OP976155 | OP976179 | N/A |
| 5 | Cape Verde | pig | G7 | HAP1 | HAP2 | HAP1 | N/A | OP976156 | OP976180 | OP976201 |
| 6 | Cape Verde | pig | G7 | HAP1 | HAP2 | HAP1 | N/A | OP976157 | OP976181 | OP976202 |
| 7 | Lithuania | pig | G7 | HAP1 | HAP2 | HAP1 | N/A | OP976158 | OP976182 | OP976203 |
| *8* | Cape Verde | dog | G7 | N/A | HAP2 | N/A | N/A | N/A | N/A | OP976204 |
| 9 | Cape Verde | dog | G7 | HAP1 | N/A | N/A | N/A | OP976159 | OP976183 | N/A |
| 10 | Cape Verde | dog | G7 | HAP1 | N/A | N/A | N/A | OP976160 | OP976184 | N/A |
| 11 | Cape Verde | dog | G7 | HAP1 | N/A | N/A | N/A | OP976161 | OP976185 | N/A |
| 12 | Cape Verde | dog | G7 | HAP1 | N/A | CV1 | N/A | OP976162 | OP976186 | N/A |
| 13 | Cape Verde | dog | G7 | HAP1 | N/A | N/A | N/A | OP976163 | OP976187 | N/A |
| 14 | Cape Verde | dog | G7 | HAP1 | N/A | N/A | N/A | OP976164 | OP976188 | N/A |
| 15 | Cape Verde | dog | G7 | HAP1 | N/A | N/A | N/A | OP976165 | OP976189 | N/A |
| 16 | Cape Verde | pig | G7 | HAP1 | N/A | N/A | N/A | OP976166 | OP976190 | N/A |
| 17 | Cape Verde | pig | G7 | HAP1 | HAP2 | N/A | N/A | OP976167 | OP976191 | OP976205 |
| 18 | Cape Verde | pig | G7 | HAP1 | N/A | N/A | N/A | OP976168 | OP976192 | N/A |
| 19 | Cape Verde | pig | G7 | HAP1 | N/A | N/A | N/A | OP976169 | OP976193 | N/A |
| 20 | Cape Verde | pig | G7 | HAP1 | N/A | N/A | N/A | OP976170 | OP976194 | N/A |
| 21 | Cape Verde | pig | G7 | HAP1 | N/A | N/A | N/A | OP976171 | OP976195 | N/A |
| 22 | Cape Verde | pig | G7 | HAP1 | N/A | N/A | N/A | OP976172 | OP976196 | N/A |
| GenBank reference | Italy | wild boar | G7 | ITA5 | N/A | N/A | N/A | MK682617 | MK682636 | N/A |
| GenBank reference | Italy | wild boar | G7 | ITA5 | N/A | N/A | N/A | MK682618 | MK682637 | N/A |
| GenBank reference | Italy | wild boar | G7 | ITA5 | N/A | N/A | N/A | MK682619 | MK682638 | N/A |
| GenBank reference | Italy | wild boar | G7 | ITA5 | N/A | N/A | N/A | MK682620 | MK682639 | N/A |
| GenBank reference | Italy | wild boar | G7 | ITA5 | N/A | N/A | N/A | MK682621 | MK682640 | N/A |
| GenBank reference | Italy | wild boar | G7 | ITA5 | N/A | N/A | N/A | MK682622 | MK682641 | N/A |
| GenBank reference | Italy | wild boar | G7 | ITA3 | N/A | N/A | N/A | MK682623 | MK682642 | N/A |
| GenBank reference | Italy | wild boar | G7 | ITA3 | N/A | N/A | N/A | MK682624 | MK682643 | N/A |
| GenBank reference | Italy | wild boar | G7 | ITA3 | N/A | N/A | N/A | MK682625 | MK682644 | N/A |
| GenBank reference | Italy | wild boar | G7 | ITA4 | N/A | N/A | N/A | MK682626 | MK682645 | N/A |
| GenBank reference | Italy | wild boar | G7 | ITA2 | N/A | N/A | N/A | MK682627 | MK682646 | N/A |
| GenBank reference | Italy | wild boar | G7 | ITA1 | N/A | N/A | N/A | MK682628 | MK682647 | N/A |
| GenBank reference | China (Tibet) | human | G7 | N/A | HAP1 | N/A | N/A | N/A | N/A | MH050631 |
| GenBank reference | France | pig | G7 | N/A | FRA2 | N/A | N/A | N/A | N/A | KX010890 |
| GenBank reference | Namibia | oryx, sheep | G7 | N/A | NAM1 | N/A | N/A | N/A | N/A | KX010889 |
| GenBank reference | Sudan | camel | G7 | N/A | HAP3 | N/A | N/A | N/A | N/A | KX010886 |
| GenBank reference | Armenia | pig | G7 | N/A | ARM1 | N/A | N/A | N/A | N/A | KX231668 |
| GenBank reference | Serbia | pig | G7 | N/A | SER3 | N/A | N/A | N/A | N/A | KX010902 |
| GenBank reference | Serbia | pig | G7 | N/A | SER2 | N/A | N/A | N/A | N/A | KX010900 |
| GenBank reference | Serbia | pig | G7 | N/A | SER1 | N/A | N/A | N/A | N/A | KX010899 |
| GenBank reference | Hungary | pig | G7 | N/A | HUN2 | N/A | N/A | N/A | N/A | KX010898 |
| GenBank reference | Hungary | pig | G7 | N/A | HUN1 | N/A | N/A | N/A | N/A | KX010897 |
| GenBank reference | Slovakia | pig | G7 | N/A | HAP4 | N/A | N/A | N/A | N/A | KX010896 |
| GenBank reference | Slovakia | pig | G7 | N/A | SVK3 | N/A | N/A | N/A | N/A | KX010894 |
| GenBank reference | Slovakia | pig | G7 | N/A | SVK1 | N/A | N/A | N/A | N/A | KX010893 |
| GenBank reference | Slovakia | pig | G7 | N/A | SVK2 | N/A | N/A | N/A | N/A | KX010892 |
| GenBank reference | France | pig | G7 | N/A | FRA1 | N/A | N/A | N/A | N/A | KX010891 |
| GenBank reference | Iran | human | G6 | N/A | N/A | N/A | N/A | N/A | N/A | MZ927665 |
| GenBank reference | Kenya | camel | G6 | N/A | N/A | N/A | N/A | N/A | N/A | MT525967 |
| GenBank reference | Argentina | pig | G7 | N/A | ARG1 | N/A | N/A | N/A | N/A | KT363812 |
| GenBank reference | China | sheep | G6 | N/A | N/A | N/A | MN340039 | N/A | N/A | N/A |
| GenBank reference | China | yak | G6 | N/A | N/A | N/A | MN340038 | N/A | N/A | N/A |
| GenBank reference | Nigeria | camel | G6 | N/A | N/A | N/A | MT166290 | N/A | N/A | N/A |
| GenBank reference | Nigeria | cattle | G6 | N/A | N/A | N/A | MT166289 | N/A | N/A | N/A |
| GenBank reference | Nigeria | camel | G6 | N/A | N/A | N/A | MT166288 | N/A | N/A | N/A |
| GenBank reference | Nigeria | camel | G6 | N/A | N/A | N/A | MT166287 | N/A | N/A | N/A |
| GenBank reference | Nigeria | camel | G6 | N/A | N/A | N/A | MT166286 | N/A | N/A | N/A |
| GenBank reference | Sudan | sheep | G6 | N/A | N/A | N/A | MH300952 | N/A | N/A | N/A |
| GenBank reference | Sudan | camel | G6 | N/A | N/A | N/A | MH300939 | N/A | N/A | N/A |
| GenBank reference | Sudan | camel | G6 | N/A | N/A | N/A | MH300950 | N/A | N/A | N/A |
| GenBank reference | Sudan | sheep | G6 | N/A | N/A | N/A | MH300940 | N/A | N/A | N/A |
| GenBank reference | Sudan | sheep | G6 | N/A | N/A | N/A | MH300941 | N/A | N/A | N/A |
| GenBank reference | Sudan | sheep | G6 | N/A | N/A | N/A | MH300942 | N/A | N/A | N/A |
| GenBank reference | Sudan | goat | G6 | N/A | N/A | N/A | MH300943 | N/A | N/A | N/A |
| GenBank reference | Sudan | goat | G6 | N/A | N/A | N/A | MH300951 | N/A | N/A | N/A |
| GenBank reference | Sudan | cattle | G6 | N/A | N/A | N/A | MH300944 | N/A | N/A | N/A |
| GenBank reference | Sudan | cattle | G6 | N/A | N/A | N/A | MH300945 | N/A | N/A | N/A |
| GenBank reference | Sudan | cattle | G6 | N/A | N/A | N/A | MH300946 | N/A | N/A | N/A |
| GenBank reference | Sudan | sheep | G6 | N/A | N/A | N/A | MH300947 | N/A | N/A | N/A |
| GenBank reference | Sudan | sheep | G6 | N/A | N/A | N/A | MH300948 | N/A | N/A | N/A |
| GenBank reference | Sudan | goat | G6 | N/A | N/A | N/A | MH300949 | N/A | N/A | N/A |
| GenBank reference | Kenya, Turkana | human | G6 | N/A | N/A | N/A | MH300936 | N/A | N/A | N/A |
| GenBank reference | Kenya, Turkana | human | G6 | N/A | N/A | N/A | MH300938 | N/A | N/A | N/A |
| GenBank reference | Kenya, Turkana | human | G6 | N/A | N/A | N/A | MH300937 | N/A | N/A | N/A |
| GenBank reference | Iran | camel | G6 | N/A | N/A | N/A | MH300931 | N/A | N/A | N/A |
| GenBank reference | Iran | camel | G6 | N/A | N/A | N/A | MH300930 | N/A | N/A | N/A |
| GenBank reference | Iran | camel | G6 | N/A | N/A | N/A | MH300932 | N/A | N/A | N/A |
| GenBank reference | Iran | camel | G6 | N/A | N/A | N/A | MH300929 | N/A | N/A | N/A |
| GenBank reference | Argentina | goat | G6 | N/A | N/A | N/A | MH300933 | N/A | N/A | N/A |
| GenBank reference | Argentina | goat | G6 | N/A | N/A | N/A | MH300934 | N/A | N/A | N/A |
| GenBank reference | Argentina | goat | G6 | N/A | N/A | N/A | MH300935 | N/A | N/A | N/A |
| GenBank reference | Mauritania | camel | G6 | N/A | N/A | N/A | MH300953 | N/A | N/A | N/A |
| GenBank reference | Mauritania | camel | G6 | N/A | N/A | N/A | MH300954 | N/A | N/A | N/A |
| GenBank reference | Poland | pig | G7 | HAP3 | HAP1 | HAP3 | AB235847 | N/A | N/A | N/A |
| GenBank reference | Argentina | pig | G7 | HAP3 | HAP1 | HAP3 | MH300969 | N/A | N/A | N/A |
| GenBank reference | Serbia | pig | G7 | SER1 | SER1 | SER1 | MH300984 | N/A | N/A | N/A |
| GenBank reference | Mexico | pig | G7 | MEX2 | HAP3 | MEX2 | MH300972 | N/A | N/A | N/A |
| GenBank reference | Mexico | pig | G7 | HAP4 | MEX1 | MEX3 | MH300979 | N/A | N/A | N/A |
| GenBank reference | Mexico | pig | G7 | HAP2 | HAP3 | HAP2 | MH300978 | N/A | N/A | N/A |
| GenBank reference | Mexico | pig | G7 | MEX2 | HAP3 | MEX2 | MH300974 | N/A | N/A | N/A |
| GenBank reference | Mexico | pig | G7 | MEX1 | HAP4 | MEX1 | MH300981 | N/A | N/A | N/A |
| GenBank reference | Mexico | pig | G7 | HAP2 | HAP3 | HAP2 | MH300975 | N/A | N/A | N/A |
| GenBank reference | Mexico | pig | G7 | HAP2 | HAP3 | HAP2 | MH300976 | N/A | N/A | N/A |
| GenBank reference | Mexico | pig | G7 | HAP2 | HAP3 | HAP2 | MH300977 | N/A | N/A | N/A |
| GenBank reference | Mexico | pig | G7 | HAP4 | MEX1 | MEX3 | MH300980 | N/A | N/A | N/A |
| GenBank reference | Mexico | pig | G7 | MEX2 | HAP3 | MEX2 | MH300973 | N/A | N/A | N/A |
| GenBank reference | Romania | sheep | G7 | HAP4 | HAP4 | HAP4 | MH300983 | N/A | N/A | N/A |
| GenBank reference | Romania | sheep | G7 | HAP2 | HAP3 | HAP2 | MH300982 | N/A | N/A | N/A |
| GenBank reference | Spain | pig | G7 | SPA1 | HAP3 | SPA1 | MH300985 | N/A | N/A | N/A |
| GenBank reference | Argentina | pig | G7 | HAP2 | HAP3 | HAP2 | MH300959 | N/A | N/A | N/A |
| GenBank reference | Argentina | pig | G7 | HAP2 | HAP3 | HAP2 | MH300960 | N/A | N/A | N/A |
| GenBank reference | Argentina | pig | G7 | HAP3 | HAP1 | HAP3 | MH300968 | N/A | N/A | N/A |
| GenBank reference | Argentina | pig | G7 | HAP2 | HAP3 | HAP2 | MH300962 | N/A | N/A | N/A |
| GenBank reference | Argentina | pig | G7 | HAP3 | HAP1 | HAP3 | MH300970 | N/A | N/A | N/A |
| GenBank reference | Argentina | pig | G7 | HAP2 | HAP3 | HAP2 | MH300963 | N/A | N/A | N/A |
| GenBank reference | Argentina | pig | G7 | HAP2 | HAP3 | HAP2 | MH300965 | N/A | N/A | N/A |
| GenBank reference | Argentina | pig | G7 | HAP2 | HAP3 | HAP2 | MH300961 | N/A | N/A | N/A |
| GenBank reference | Argentina | pig | G7 | HAP2 | HAP3 | HAP2 | MH300967 | N/A | N/A | N/A |
| GenBank reference | Argentina | pig | G7 | HAP2 | HAP3 | HAP2 | MH300957 | N/A | N/A | N/A |
| GenBank reference | Argentina | pig | G7 | HAP2 | HAP3 | HAP2 | MH300964 | N/A | N/A | N/A |
| GenBank reference | Argentina | pig | G7 | HAP2 | HAP3 | HAP2 | MH300966 | N/A | N/A | N/A |
| GenBank reference | Argentina | pig | G7 | HAP2 | HAP3 | HAP2 | MH300958 | N/A | N/A | N/A |
| GenBank reference | Argentina | pig | G7 | HAP2 | HAP3 | HAP2 | MH300955 | N/A | N/A | N/A |
| GenBank reference | France (Corsica) | pig | G7 | FRA2 | FRA2 | FRA2 | MH300986 | N/A | N/A | N/A |
| GenBank reference | France (Corsica) | pig | G7 | FRA3 | FRA2 | FRA3 | MH300995 | N/A | N/A | N/A |
| GenBank reference | France (Corsica) | pig | G7 | FRA3 | FRA2 | FRA3 | MH300996 | N/A | N/A | N/A |
| GenBank reference | France (Corsica) | pig | G7 | FRA3 | FRA2 | FRA3 | MH300997 | N/A | N/A | N/A |
| GenBank reference | France (Corsica) | pig | G7 | FRA1 | FRA2 | FRA1 | MH300989 | N/A | N/A | N/A |
| GenBank reference | France (Corsica) | pig | G7 | FRA1 | FRA2 | FRA1 | MH300987 | N/A | N/A | N/A |
| GenBank reference | France (Corsica) | pig | G7 | FRA3 | FRA2 | FRA3 | MH300999 | N/A | N/A | N/A |
| GenBank reference | France (Corsica) | pig | G7 | FRA3 | FRA2 | FRA3 | MH300998 | N/A | N/A | N/A |
| GenBank reference | France (Corsica) | pig | G7 | FRA3 | FRA2 | FRA3 | MH300992 | N/A | N/A | N/A |
| GenBank reference | France (Corsica) | pig | G7 | FRA3 | FRA2 | FRA3 | MH301002 | N/A | N/A | N/A |
| GenBank reference | France (Corsica) | pig | G7 | FRA1 | FRA2 | FRA1 | MH300990 | N/A | N/A | N/A |
| GenBank reference | France (Corsica) | pig | G7 | FRA1 | FRA2 | FRA1 | MH300991 | N/A | N/A | N/A |
| GenBank reference | France (Corsica) | pig | G7 | FRA3 | FRA2 | FRA3 | MH300993 | N/A | N/A | N/A |
| GenBank reference | France (Corsica) | pig | G7 | FRA3 | FRA2 | FRA3 | MH301000 | N/A | N/A | N/A |
| GenBank reference | France (Corsica) | pig | G7 | FRA3 | FRA2 | FRA3 | MH301001 | N/A | N/A | N/A |
| GenBank reference | France (Corsica) | pig | G7 | FRA3 | FRA2 | FRA3 | MH300994 | N/A | N/A | N/A |
| GenBank reference | France (Corsica) | pig | G7 | FRA1 | FRA2 | FRA3 | MH300988 | N/A | N/A | N/A |
| GenBank reference | Poland | human | G7 | HAP1 | HAP2 | HAP1 | MH301003 | N/A | N/A | N/A |
| GenBank reference | Poland | human | G7 | POL1 | POL1 | POL1 | MH301007 | N/A | N/A | N/A |
| GenBank reference | Poland | human | G7 | HAP1 | HAP2 | HAP1 | MH301005 | N/A | N/A | N/A |
| GenBank reference | Ukraine | pig | G7 | UKR1 | HAP4 | UKR1 | MH301022 | N/A | N/A | N/A |
| GenBank reference | Poland | pig | G7 | HAP1 | HAP2 | HAP1 | MH301004 | N/A | N/A | N/A |
| GenBank reference | Lithuania | pig | G7 | LIT1 | HAP2 | LIT1 | MH301020 | N/A | N/A | N/A |
| GenBank reference | Poland | pig | G7 | HAP4 | POL2 | HAP4 | MH301006 | N/A | N/A | N/A |
| GenBank reference | Ukraine | pig | G7 | UKR1 | HAP4 | UKR1 | MH301021 | N/A | N/A | N/A |
| GenBank reference | Italy (Sardinia) | pig | G7b | N/A | N/A | N/A | MH301018 | N/A | N/A | N/A |
| GenBank reference | Italy (Sardinia) | pig | G7b | N/A | N/A | N/A | MH301019 | N/A | N/A | N/A |
| GenBank reference | France (Corsica) | pig | G7b | N/A | N/A | N/A | MH301010 | N/A | N/A | N/A |
| GenBank reference | France (Corsica) | pig | G7b | N/A | N/A | N/A | MH301008 | N/A | N/A | N/A |
| GenBank reference | France (Corsica) | pig | G7b | N/A | N/A | N/A | MH301014 | N/A | N/A | N/A |
| GenBank reference | France (Corsica) | pig | G7b | N/A | N/A | N/A | MH301011 | N/A | N/A | N/A |
| GenBank reference | France (Corsica) | pig | G7b | N/A | N/A | N/A | MH301012 | N/A | N/A | N/A |
| GenBank reference | France (Corsica) | pig | G7b | N/A | N/A | N/A | MH301013 | N/A | N/A | N/A |
| GenBank reference | France (Corsica) | pig | G7b | N/A | N/A | N/A | MH301015 | N/A | N/A | N/A |
| GenBank reference | France (Corsica) | pig | G7b | N/A | N/A | N/A | MH301009 | N/A | N/A | N/A |
| GenBank reference | France (Corsica) | pig | G7b | N/A | N/A | N/A | MH301016 | N/A | N/A | N/A |
| *Identical sequence from different cysts from the same host. Not included in the phylogenetic network analysis.  N/A – haplotype name not assigned, or data missing/not included. | | | | | | | | | | |

Table S3: Sample ID, origin (island, district, city, local and place of collection), host species, sample type and condition and sequencing results for Taenia and Hydatigera positive samples identified from tissue cysts or pathological lesions and faecal samples in Cape Verde.

| **Sample ID** | **Origin (Island)** | **District, City, Local** | **Place of collection** | **Host*, Organ** | **Sample type** | **Sequencing results** |
| --- | --- | --- | --- | --- | --- | --- |
| T1 | Santiago | Santa Catarina, Assomada | Livestock Market | Goat, liver | Cysts/lesions** | *Taenia hydatigena* |
| T2 | Santiago | Santa Catarina, Assomada | Municipal market/butcher | Cattle, heart | Cysts/lesions** | *T. hydatigena* |
| T3 | Santiago | Santa Catarina, Assomada | Slaughterhouse | Cattle, liver | Cysts/lesions** | *T. hydatigena* |
| T4 | Santiago | Santa Catarina, Assomada | Livestock Market | Sheep, *omentum* | Cyst | *T. hydatigena* |
| T5 | Santiago | Santa Catarina, Assomada | Slaughterhouse | Cattle, liver | Cyst | *T. hydatigena* |
| T6 | Santiago | Santa Catarina, Assomada | Municipal market/butcher | Pig, liver | Cyst | *T. hydatigena* |
| T7 | Santiago | Santa Catarina, Assomada | Municipal market/butcher | Pig, liver | Cysts/lesions** | *T. hydatigena* |
| T8 | Santiago | Santa Catarina, Assomada | Municipal market/butcher | Pig, liver | Cysts/lesions** | *T. hydatigena* |
| T9 | Santiago | Santa Catarina, Assomada | Municipal market/butcher | Pig, liver | Cysts/lesions** | *T. hydatigena* |
| T10 | Santiago | Santa Catarina, Assomada | Slaughterhouse | Cattle, liver | Cysts/lesions** | *T. hydatigena* |
| T11 | Santiago | Santa Catarina, Assomada, Cumben | Municipal market/butcher | Pig, liver | Cysts/lesions** | *T. hydatigena* |
| T12 | Santiago | Santa Catarina, Assomada, Cumben | Municipal market/butcher | Pig, liver | Cysts/lesions** | *T. hydatigena* |
| T13 | Santiago | Praia, Praia, Achada São Filipe | Slaughterhouse | Pig, *omentum* | Cysts/lesions** | *T. hydatigena* |
| T14 | Sal | Sal, Espargos, City Center | Slaughterhouse | Pig, liver | Cyst | *Taenia* sp*.* |
| T15 | Boavista | Boavista, Sal Rei | Home slaughter spot | Pig, *omentum* | Cysts | *T. hydatigena* |
| T16 | Boavista | Boavista, Rabil | Home slaughter spot | Pig, *omentum* | Cysts/lesions** | *T. hydatigena* |
| T17 | Santiago | Santa Catarina, Assomada, Chão Santo | Around home slaughter spot | Dog | Faecal | *Hydatigera taeniaeformis* |
| T18 | Santiago | Santa Catarina, Assomada, Cruz Grande | Around home slaughter spot | Dog | Faecal | *T. hydatigena* |
| T19 | Santiago | Praia, Praia, Achada São Filipe | Around Slaughterhouse | Dog | Faecal | *Taenia* sp*.* |
| T20 | Santiago | Praia, Praia, Achada Grande Frente | Around home slaughter spot | Dog | Faecal | *T. hydatigena* |
| T21 | Santiago | Praia, Praia, Achada Grande Frente | Around home slaughter spot | Dog | Faecal | *T. hydatigena* |
| T22 | Santiago | Praia, Praia, Achada Grande Frente | Around home slaughter spot | Dog | Faecal | *T. hydatigena* |
| T23 | Santiago | Praia, Praia, Várzea | Around home slaughter spot | Dog | Faecal | *T. hydatigena* |
| T24 | Santiago | Praia, Praia, Fazenda | Around Livestock Market | Dog | Faecal | *T. hydatigena* |
| T25 | Santiago | Praia, Praia, Fazenda | Around Livestock Market | Dog | Faecal | *T. hydatigena* |
| T26 | Santiago | Praia, Praia, Fazenda | Around Livestock Market | Dog | Faecal | *T. hydatigena* |
| T27 | Santiago | Praia, Praia, Fazenda | Around Livestock Market | Dog | Faecal | *T. hydatigena* |
| T28 | Santiago | Praia, Praia, Fazenda | Around Livestock Market | Dog | Faecal | *T. hydatigena* |
| T29 | Santiago | Praia, Praia, Achadinha | Around home slaughter spot | Dog | Faecal | *T. hydatigena* |
| T30 | Santiago | Santa Cruz, Achada Fazenda | Around home slaughter spot | Dog | Faecal | *T. hydatigena* |
| T31 | Santiago | Santa Cruz, Achada Laje | Around home slaughter spot | Dog | Faecal | *T. hydatigena* |
| T32 | Santiago | São Miguel, Calheta | Around Slaughterhouse | Dog | Faecal | *T. hydatigena* |
| T33 | Santiago | Praia, Praia, Achada São Filipe | Around Slaughterhouse | Dog | Faecal | *T. hydatigena* |
| T34 | Fogo | Mosteiros, Mosteiros | Around Slaughterhouse | Dog | Faecal | *T. hydatigena* |
| T35 | Fogo | Mosteiros, Queimada Guincho | Around home slaughter spot | Dog | Faecal | *T. hydatigena* |
| T36 | Boavista | Boavista, Sal Rei | Around home slaughter spot | Dog | Faecal | *T. hydatigena* |
| T37 | São Vicente | São Vicente, Km6 | Around home slaughter spot | Dog | Faecal | *T. hydatigena* |
| T38 | São Vicente | São Vicente, Km6 | Around home slaughter spot | Dog | Faecal | *T. hydatigena* |
| T39 | Santo Antão | Ribeira Grande | Around Slaughterhouse | Dog | Faecal | *T. hydatigena* |
| T40 | Santo Antão | Ribeira Grande | Around home slaughter spot | Dog | Faecal | *T. hydatigena* |
| * faecal samples of dogs: in few cases cat or human origin cannot be excluded  ** tissue cysts or pathological lesions without cyst formation (No information available concerning the structure of the sample) | | | | | |  |


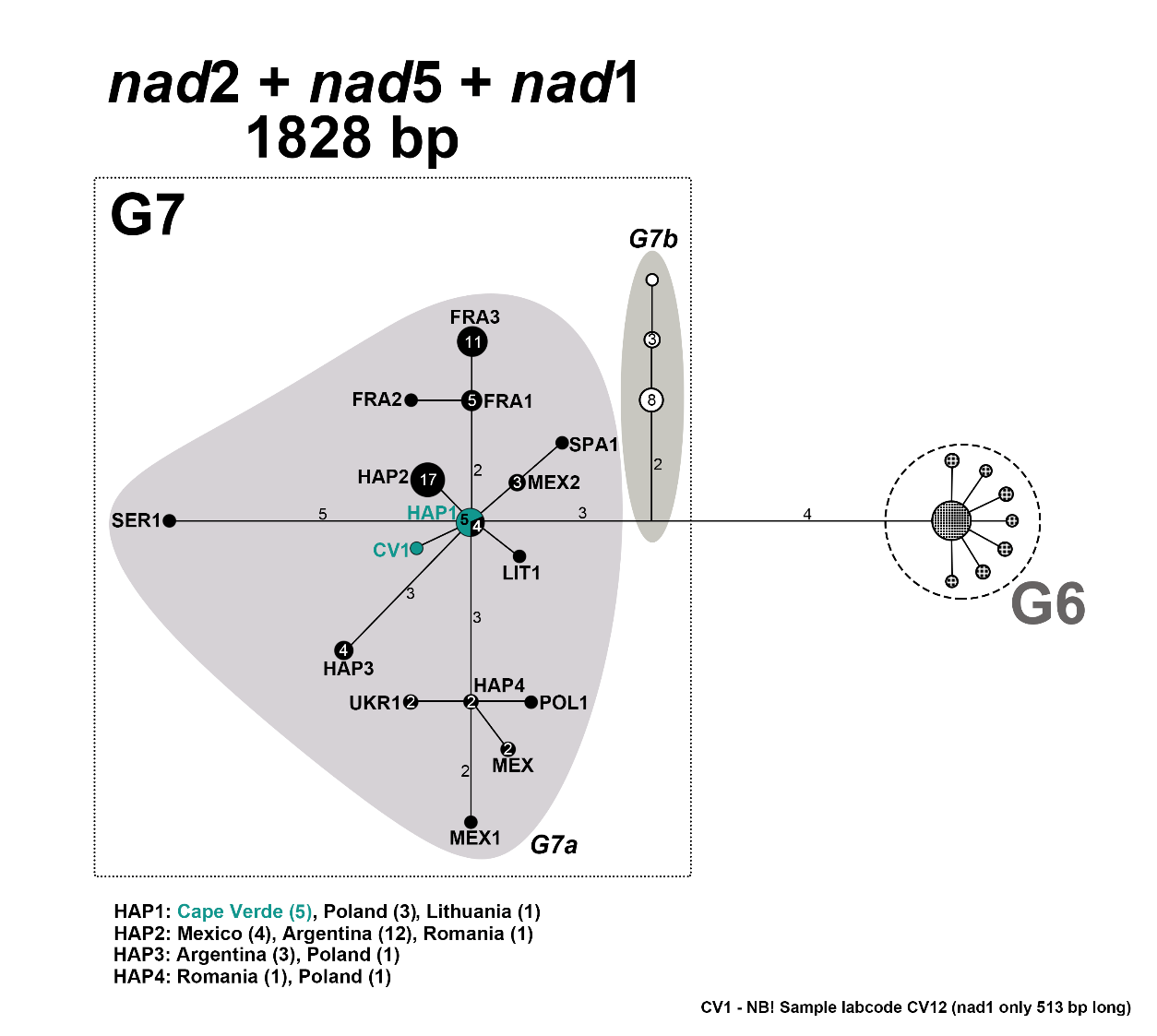


Figure S1 - Median-joining network of Echinococcus granulosus s.l. G7 isolates from Cape Verde and respective G6-G7 reference sequences from GenBank based on concatenated NADH dehydrogenase subunit one (nad1, 513bp), two (nad2, 701bp) and five (nad5, 614bp) gene fragments. Cape Verde sequences of the current study obtained from tissue (n=6) and faecal samples (n=1) are depicted in green. References from GenBank of genotype G6 and haplogroup G7b are represented schematically, and references of G7 are depicted in black. Numbers inside the circles represent the number of identical sequences within the respective haplotype; numbers beside the lines represent the number of mutations. Haplotype names are designated as two or three letter abbreviations (HAP – haplotypes representing samples originating from different countries, CV – Cape Verde, FRA- France (Corsica), LIT – Lithuania, MEX – Mexico, POL – Poland, SER – Serbia, SPA – Spain, SVK – Slovakia, UKR – Ukraine).
